# Novel high-performance microstrip diplexer for 5G mid-band and wide-band applications: Design, analysis and manufacturing

**DOI:** 10.1371/journal.pone.0327839

**Published:** 2025-07-11

**Authors:** Salah I. Yahya, Farid Zubir, Mohammed Abdel Hafez, Leila Nouri, Muhammad Akmal Chaudhary, Maher Assaad, Abbas Rezaei, Noorlindawaty Md Jizat

**Affiliations:** 1 Department of Communication and Computer Engineering, Cihan University-Erbil, Erbil, Iraq; 2 Department of Software Engineering, Faculty of Engineering, Koya University, Koya, Iraq; 3 Wireless Communication Centre, Faculty of Electrical Engineering, Universiti Teknologi Malaysia, Johor Bahru, Johor, Malaysia; 4 Department of Electrical and Communication Engineering, United Arab Emirates University, Al Ain, United Arab Emirates; 5 Institute of Research and Development, Duy Tan University, Da Nang, Vietnam; 6 School of Engineering & Technology, Duy Tan University, Da Nang, Vietnam; 7 Department of Electrical and Computer Engineering, College of Engineering and Information Technology, Ajman University, Ajman, United Arab Emirates; 8 Department of Electrical Engineering, Kermanshah University of Technology, Kermanshah, Iran; 9 Faculty of Engineering, Multimedia University, Persiaran Multimedia, Cyberjaya, Selangor, Malaysia; Information Technology University, PAKISTAN

## Abstract

This paper presents the design and experimental results of a microstrip diplexer with a high performance for 5G applications. The introduced diplexer has compact size, novel structure, low losses, and wide fractional bandwidth. Notably, it exhibits a novel microstrip layout with a very compact size of 0.004 λ_g_^2^. The resonance frequencies are tuned at 1.1 GHz and 3.2 GHz for mid-band 5G applications. The presented structure has the fractional bandwidths (57.3%, 44.6%) and insertion losses (0.07 dB, 0.04 dB). Additionally, it features two flat channels with two low maximum group delays of 0.86 ns, 0.4 ns in the 1^st^ and 2^nd^ passbands, respectively. A perfect mathematical design method is applied to find the behavior of the introduced resonator, as well as the most effective physical dimensions. For improving the performance and miniaturization, an optimization method is used. To validate the design approach, the proposed diplexer is fabricated and then measured, demonstrating a close agreement between the simulation and measurement results. This highlights the effectiveness of our design approach and underscores the potential of the proposed diplexer for enabling efficient and reliable communication in the rapidly evolving field of telecommunications.

## Introduction

Designing high-performance devices for 5G communication application characterized by low loss, compact size, low group delay, and high isolation is of utmost importance in the rapidly evolving field of telecommunications [1–6]. In the era of 5G, where data rates are expected to reach unprecedented levels, the demand for efficient and reliable communication systems is skyrocketing. Microstrip technology is an efficient way to design telecommunications devices [7–9]. Some microstrip devices are reported in [10–13] for communication systems. Microstrip diplexers play a crucial role in enabling simultaneous transmission and reception of multiple frequency bands. This maximizes the utilization of available spectrum resources enabling the coexistence of various wireless technologies like Wi-Fi, Bluetooth, and cellular networks. By increasing the number of frequency bands and the need for multi-band operation, it is crucial to minimize the physical size of diplexers. Low loss is another critical requirement for a high-performance diplexer. By minimizing insertion loss, the diplexer ensures efficient signal transmission and reception, leading to improved enhanced user experience and data rates. In addition to low loss, low group delay is imperative for maintaining signal integrity. High group delay can lead to distortion and degradation of signals, especially in applications where timing is critical. Hence, the design of a diplexer with low group delay is essential to ensure precise and reliable signal transmission.

In recent times, microstrip passive filtering devices have been widely used in mid-band 5G communication systems. Various types of dual-band Bandpass-Bandpass (BP-BP) diplexers have been proposed in [14–25]. Coupled step impedance open loops [10], the mixed-mode triangular substrate [15], similar four coupled lines [16,17] and coupled close loops [18] are used to obtain some microstrip diplexers. A narrow passband may greatly increase the group delay. Therefore, the use of this type of diplexers may be practically impossible. However, the designed diplexers in [14–18] encounter issues with narrow channels despite occupying large areas. The proposed diplexers in [19,20] are characterized by their large size, high insertion losses and narrow channels. A large-size microstrip diplexer is presented in [21] for the worldwide interoperability of Wireless Local Area Networks (WLANs) and Worldwide Interoperability for Microwave Access (WiMAX). Notably, all reported diplexers in [22–26] fail to address the issues related to large size adequately and do not significantly enhance fractional bandwidths. In [24], the step impedance unit-cell is employed to create a microstrip diplexer. However, the diplexer designed in [24] exhibits high insertion losses at both lower and upper channels. The proposed diplexer in [25] is designed for the Global System for Mobile Communications (GSM) and Long-Term Evolution (LTE) applications.

In this paper, using a novel microstrip configuration we will design a diplexer for mid-band 5G applications encompassing the frequency range from 1 GHz up to 6 GHz. The proposed diplexer has many advantages in terms of low insertion loss, very compact size, wide fractional bandwidths, low group delays and flat passbands. Its wide passbands make it suitable for application in wide-band communication systems.

Therefore, the goal is to design a microstrip diplexer with the following advantages in comparison with the previous works:

- Novel structure with very compact size
- The widest FBWs
- The lowest insertion losses
- Low group delays
- Flat passbands
- Negative group delay
- Good return loss and isolation

The design process is initiated with the presentation and mathematical analysis of a novel resonator. Then, two microstrip bandpass filters will be designed using this resonator. Finally, by connecting these filters, our diplexer will be completed. Using a mathematical analysis, we will determine the behavior of the proposed resonator and identify the most effective dimensions affecting its frequency response. Therefore, it can be optimized easily to improve the performance and miniaturization simultaneously. This work introduces a novel microstrip resonator configuration specifically designed for mid-band 5G applications. This diplexer features significant advancements, including a highly compact size, improved fractional bandwidths and notably low insertion losses. Additionally, the implementation of flat passbands and negative group delays represents a substantial innovation over the previous designs. These enhancements not only demonstrate the versatility of our design approach but also underscore the potential of the proposed diplexer in meeting the demands of modern telecommunications systems.

## Design of the proposed structures

**Fig 1:**
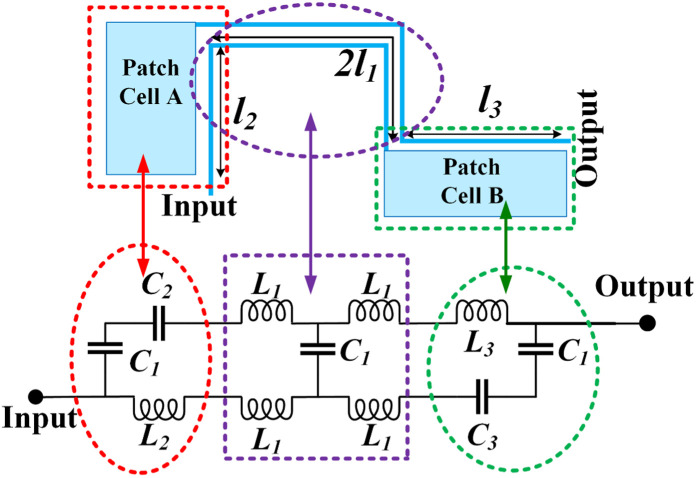
Basic resonator with its equivalent LC circuit.

**Fig.2:**
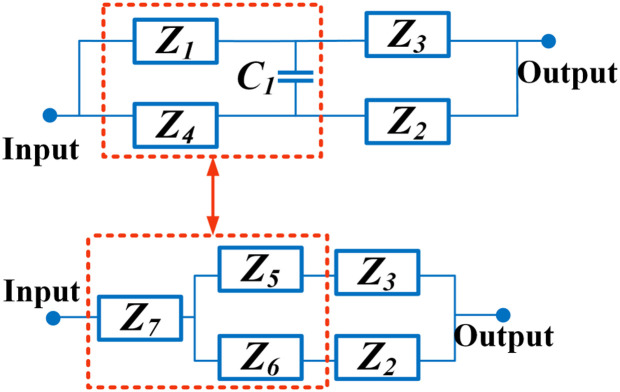
Simplified equivalent circuit.


Our method to achieve a microstrip diplexer is to design two bandpass filters (BPFs) working at different frequencies. Then, we will connect these filters together. To obtain both filters, first we will design only one basic resonator. Then, this basic resonator will be upgraded to the considered filters by tuning its resonance frequency and improving its frequency responses. Coupled lines play a crucial role in the construction of bandpass resonators. Coupled lines act as the capacitive metal plates filled with dielectric. Therefore, the equivalent circuit of coupled lines includes coupling capacitors C_1_. Since these lines are thin, they have inductive feature. Consequently, the approximated model of a pair of coupled lines includes four inductors and one capacitor [23]. To obtain a more exact model, we have to increase the number of inductors and capacitors as a ladder network. If the number of inductors is more the model will be more accurate. By shifting the resonance frequency to the left, the patch cells save the overall size significantly. Our basic resonator is proposed in Fig 1. To further reduce the size, spiral coupled lines are employed [23]. An approximated LC circuit of the proposed resonator is presented in Fig 1. The approximated models of three sections are marked. The equivalent of thin coupled lines with *2l*_*1*_ lengths includes the inductors *L*_*1*_ and a coupling capacitor (*C*_*1*_). Note that we replaced a physical length of *l*_*1*_ with an inductor of *L*_*1*_. The patch Cell A (coupled to the physical length *l*_*2*_) is replaced by a coupling capacitor of *C*_*1*_, an inductor of *L*_*2*_ and a capacitor of *C*_*2*_, where, *C*_*2*_ is the patch Cell A equivalent. As can be seen in Fig 1, a coupling capacitor is in series with C_2_ and another coupling capacitor is in series with C_3_. The lengths *l*_*2*_ and *l*_*3*_ are shown by *L*_*2*_ and *L*_*3*_ inductors. Moreover, the equivalent of patch cell B, is the capacitor *C*_*3*_. In the coupled lines exact model, the number of coupling capacitors is more. The capacitance effects between the transmission lines and the ground layer are negligible at frequencies below 10 GHz. However, at higher frequencies, these capacitance effects can disrupt the frequency response. The simplified equivalent circuits of the presented resonator are depicted in Fig 2, where the impedances are defined as follows:

$$Z_{1}=\frac{1}{j\omega C_{1}}+\frac{1}{j\omega C_{2}}+j\omega L_{1}$$ (1)

$$Z_{2}=\frac{1}{j\omega C_{1}}+\frac{1}{j\omega C_{3}}+j\omega L_{1}$$ (2)

$$Z_{3}=j\omega L_{1}+j\omega L_{3}$$ (3)

$$Z_{4}=j\omega L_{1}+j\omega L_{2}$$ (4)

The impedances Z_5_, Z_6_ and Z_7_ are obtained by Δ to Y conversion as follows:


$$\begin{array}{c} Z_{5}=\frac{Z_{1}\times \frac{1}{j\omega C_{1}}}{Z_{1}+Z_{4}+\frac{1}{j\omega C_{1}}}\to Z_{5}=\frac{(\frac{1}{j\omega C_{1}}+\frac{1}{j\omega C_{2}}+j\omega L_{1})\times \frac{1}{j\omega C_{1}}}{\frac{1}{j\omega C_{1}}+\frac{1}{j\omega C_{2}}+j\omega L_{1}+j\omega L_{1}+j\omega L_{2}+\frac{1}{j\omega C_{1}}}\to \\ Z_{5}=\frac{C_{2}+C_{1}-\omega^{2}L_{1}C_{2}C_{1}}{j\omega C_{1}[2C_{2}+C_{1}-C_{2}C_{1}\omega^{2}(2L_{1}+L_{2})]} \end{array}$$ (5)

$$\begin{array}{c} Z_{6}=\frac{Z_{4}\times \frac{1}{j\omega C_{1}}}{Z_{1}+Z_{4}+\frac{1}{j\omega C_{1}}}\to \\ Z_{6}=\frac{(j\omega L_{1}+j\omega L_{2})\times \frac{1}{j\omega C_{1}}}{\frac{1}{j\omega C_{1}}+\frac{1}{j\omega C_{2}}+j\omega L_{1}+j\omega L_{1}+j\omega L_{2}+\frac{1}{j\omega C_{1}}}\to \\ Z_{6}=\frac{j\omega (L_{1}+L_{2})C_{2}}{2C_{2}+C_{1}-\omega^{2}(2L_{1}+L_{2})C_{1}C_{2}} \end{array}$$ (6)

$$\begin{array}{c} Z_{7}=\frac{Z_{1}\times Z_{4}}{Z_{1}+Z_{4}+\frac{1}{j\omega C_{1}}}\to \\ Z_{7}=\frac{\left(j\omega L_{1}+j\omega L_{2})\times (\frac{1}{j\omega C_{1}}+\frac{1}{j\omega C_{2}}+j\omega L_{1}\right)}{\frac{1}{j\omega C_{1}}+\frac{1}{j\omega C_{2}}+j\omega L_{1}+j\omega L_{1}+j\omega L_{2}+\frac{1}{j\omega C_{1}}}\to \\ Z_{7}=\frac{j\omega (L_{1}+L_{2})\times (C_{2}+C_{1}-\omega^{2}C_{1}C_{2}L_{1})}{2C_{2}+C_{1}-\omega^{2}C_{1}C_{2}(2L_{1}+L_{2})} \end{array}$$ (7)

where the angular frequency is ω. Capacitors (*C*_*1*_) are usually very small values in fF (or pF). As mentioned before, *C*_*1*_ is the capacitance between thin coupled lines. Meanwhile, the coupling capacitors between patch cells and thin lines are *C*_*1*_. Since the coupling capacitors are very small in both cases, this approximation is acceptable with a high accuracy. Also, the frequency target and inductors are in GHz and nH, respectively. Therefore:


$$\frac{1}{j\omega C_{1}}+\frac{1}{j\omega C_{2}}+j\omega L_{1}\approx \frac{1}{j\omega C_{1}}$$ (8)

$$\frac{1}{j\omega C_{1}}+\frac{1}{j\omega C_{3}}+j\omega L_{1}\approx \frac{1}{j\omega C_{1}}$$ (9)

$$2C_{2}+C_{1}-C_{2}C_{1}\omega^{2}(2L_{1}+L_{2})\approx 2C_{2}$$ (10)

$$C_{2}+C_{1}-\omega^{2}L_{1}C_{2}C_{1}\approx C_{2}$$ (11)

Using the above approximations, we can simplify some of the defined impedances as follows:


$$Z_{1}\approx Z_{2}\approx \frac{1}{j\omega C_{1}}$$ (12)

$$Z_{5}\approx \frac{1}{2j\omega C_{1}}$$ (13)

$$Z_{6}\approx Z_{7}\approx \frac{j\omega (L_{1}+L_{2})}{2}$$ (14)

Now we can calculate *Z*_*eq*_ (the impedance between the input and output ports) as follows:


$$\begin{array}{c} Z_{eq}=\frac{\left(Z_{5}+Z_{3})\times (Z_{2}+Z_{6}\right)}{Z_{5}+Z_{3}+Z_{2}+Z_{6}}+Z_{7}\to \\ Z_{eq}\approx \frac{\left(\frac{1}{2j\omega C_{1}}+j\omega L_{1}+j\omega L_{3})\times (\frac{1}{j\omega C_{1}}+\frac{j\omega (L_{1}+L_{2})}{2}\right)}{\frac{1}{2j\omega C_{1}}+j\omega L_{1}+j\omega L_{3}+\frac{1}{j\omega C_{1}}+\frac{j\omega (L_{1}+L_{2})}{2}}+\frac{j\omega (L_{1}+L_{2})}{2} \end{array}$$ (15)

According to the last equation, the value of the equivalent impedance is strongly dependent on the capacitor *C*_*1*_. If the distance between the coupled lines decreases, the value of this capacitor increases. According to this equivalent impedance, the resonant frequency can be determined. The structure can also be miniaturized. Because of the high degree of freedom for choosing *L*_*1*_*, L*_*2*_ and *L*_*3*_, the structure can easily be chosen in the smallest possible overall size. Using *Z*_*eq*_, we can calculate the insertion loss of the proposed resonator (*IL*_*r*_) for a terminal impedance of *Z*_*0*_ as follows:


$$IL_{r}=-20\log\left|\frac{2}{2+Z_{eq}/Z_{0}}\right|$$ (16)

According to Equation (16), to obtain a low insertion loss at a resonance frequency of *ω*_*r*_ the following conditions must be considered:


$$\begin{array}{c} IL_{r}=0\to Z_{eq}=0\to \\ \left\{ \begin{array}{c} \frac{\left(\frac{1}{2j\omega_{r}C_{1}}+j\omega_{r}(L_{1}+L_{3}))\times (\frac{1}{j\omega_{r}C_{1}}+\frac{j\omega_{r}(L_{1}+L_{2})}{2}\right)}{\frac{3}{2j\omega_{r}C_{1}}+j\omega_{r}(L_{1}+L_{3})+\frac{j\omega_{r}(L_{1}+L_{2})}{2}}=-\frac{j\omega_{r}(L_{1}+L_{2})}{2}\\ (\frac{1}{2j\omega_{r}C_{1}}+j\omega_{r}(L_{1}+L_{3}))\times (\frac{1}{j\omega_{r}C_{1}}+\frac{j\omega_{r}(L_{1}+L_{2})}{2})\approx \frac{1}{2j\omega_{r}C_{1}}\times \frac{1}{j\omega_{r}C_{1}} \end{array}\right.\;\to \\ \omega_{r}^{4}C_{1}^{2}(L_{1}+L_{2})(3L_{1}+2L_{3}+L_{2})-3\omega_{r}^{2}(L_{1}+L_{2})C_{1}+1=0\to \\ \left\{ \begin{array}{c} \omega_{r1}=\sqrt{\frac{3(L_{1}+L_{2})+\sqrt{{\left(3(L_{1}+L_{2})\right)}^{2}-4(L_{1}+L_{2})(3L_{1}+2L_{3}+L_{2})}}{2C_{1}(L_{1}+L_{2})(3L_{1}+2L_{3}+L_{2})}}\\ \omega_{r2}=\sqrt{\frac{3(L_{1}+L_{2})-\sqrt{{\left(3(L_{1}+L_{2})\right)}^{2}-4(L_{1}+L_{2})(3L_{1}+2L_{3}+L_{2})}}{2C_{1}(L_{1}+L_{2})(3L_{1}+2L_{3}+L_{2})}} \end{array}\right.\; \end{array}$$ (17)

Based on Equation (17), our resonator has two resonance frequencies *ω*_*r1*_ and *ω*_*r2*_. Our goal is to design a resonator with only one resonance frequency. Therefore, *ω*_*r2*_ is the main resonance frequency and *ω*_*r1*_ is a harmonic. To remove this harmonic, we can let *ω*_*r1*_* = ω*_*r2*_. Hence:


$$\;\;\;\sqrt{\frac{3L_{1}+2L_{3}+L_{2}}{L_{1}+L_{2}}}=1.5$$ (18)

**Fig 3:**
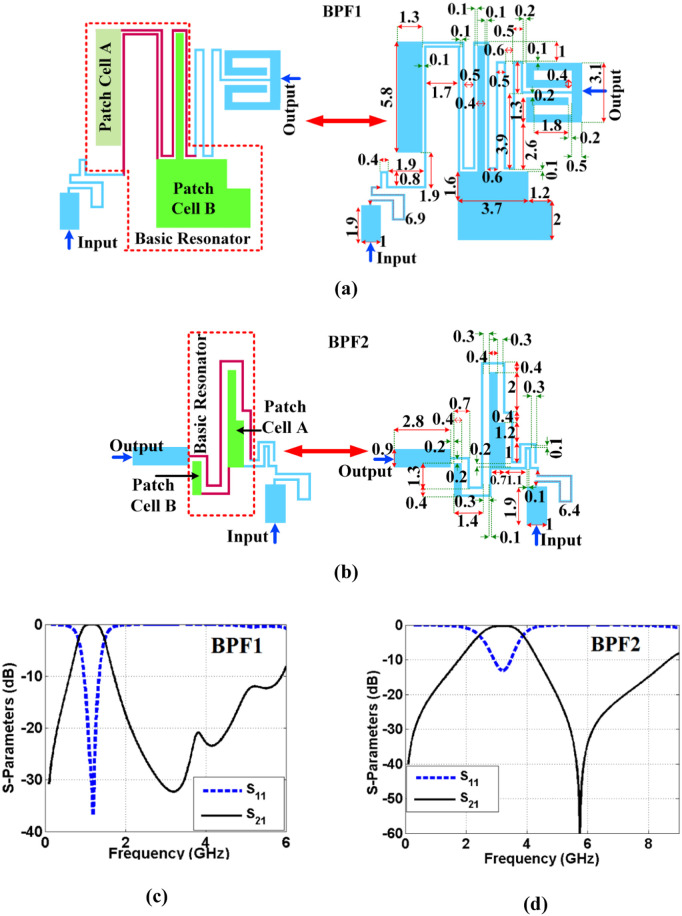
The BPFs and their frequency responses: (a) layout of BPF1, (b) layout of BPF2, (c) frequency response of BPF1, (d) frequency response of BPF2 (unit: mm).

If the ratio of inductances is adjusted based on Equation (18), this harmonic can be eliminated. However, if this leads to an increase in the size, *ω*_*r1*_ should be placed at a high frequency. According to Equation (18), if we increase the physical lengths *l*_*1*_ and *l*_*2*_, we must decrease *l*_*3*_ so that their ratio remains constant. Therefore, the above point should be taken into consideration in the optimization so that no additional harmonics are produced. Finally, we have to see which method saves more size. Using the proposed resonator, we designed two BPFs named as BPF1 and BPF2. Figs 3(a)–3(d) show the layout of BPF1, layout of BPF2, frequency response of BPF1 and frequency response of BPF2 respectively. The use of coupled thin transmission lines is the main reason for creating passbands. These lines have inductance characteristics (*L*_*1*_), but coupling capacitors (*C*_*1*_) are created between them. Placing these inductors and capacitors (*L*_*1*_ and *C*_*1*_) together creates a passband. However, patch stubs are also effective for controlling the bandwidth. They have a significant effect on the −3 dB cut-off frequencies. The simulation results show that the bandwidth becomes narrower with an increase in the size of these cells. Also, the operating frequencies shift to the left. As noted, patch stubs are smaller in BPF2 than BPF1. Therefore, this filter works at a higher frequency where it has a wider bandwidth. But increasing the size of these stubs too much leads to decrease in the frequency selectivity. We used Rogers/RT Duroid5880 substrate (*h* = 0.7874 mm, *tan(δ)* = 0.0009 and *ɛ*_*r*_ = 2.22) to design both filters. In addition, this substrate is used to design and fabricate our diplexer. The BPF1 and BPF2 work at 1.1 GHz and 3.2 GHz. For BPF1, the return and insertion losses are 36.8 dB and 0.018 dB respectively. Meanwhile, BPF2 has return and insertion losses of 13.1 dB and 0.2 dB. The passbands of both filters are flat with low group delays. In Figs 3(a) and 3(b), the structure of the analyzed resonator is specified inside the BPFs. In addition, the positions of microstrip patch cells are specified which are connected by the coupled lines. Since our goal is to achieve two filters with different resonant frequencies, the structure of patch cells in BPF1 and BPF2 is chosen differently. Other thin spiral cells are added to upgrade the resonators and improve the frequency responses based on the optimization method.

**Fig 4:**
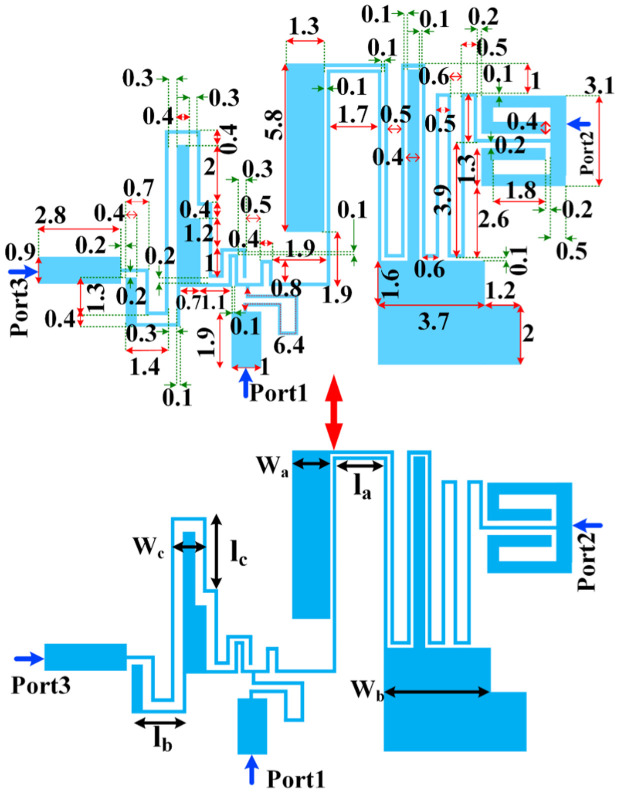
Presented diplexer.

In summary, the design process for the bandpass filters utilizing the microstrip basic resonator is systematic and involves several key steps to ensure optimal performance. The initial step involves the mathematical analysis of the equivalent LC circuit model of the microstrip resonator. By deriving specific formulas that describe the resonator’s behavior, it can be understand how various parameters influence its performance. This analysis provides insight into the relationships between physical dimensions, resonance frequency, and quality factor. The flexibility in choosing certain lengths allows optimizing the dimensions for miniaturization without compromising performance. The dimensions are selected carefully that meet the desired resonance frequency while ensuring that the bandwidth and insertion loss remain within acceptable limits. Once the resonator dimensions are established, we proceed to design the bandpass filters. These filters are designed directly from the resonator characteristics, leveraging its frequency response to achieve the desired passband. The coupling between resonators is also considered during this phase to ensure that the filters exhibit the required selectivity and rejection levels. After an initial design is completed, electromagnetic simulations are performed to validate the theoretical predictions. This iterative process allows for adjustments in dimensions and configurations to fine-tune performance metrics such as return loss, insertion loss, and bandwidth. By integrating the proposed BPFs, a microstrip diplexer with a size of 18.3 mm × 10.5 mm (0.09 λ_g_ × 0.05 λ_g_) is obtained. The parameter λ_g_, is guided wave length calculated at 1.1 GHz (the first operating frequency). This diplexer is presented in Fig 4. Thin meander lines and open stubs are suitable choices for miniaturizing and improving the frequency response [27,28]. Therefore, we added them to the analyzed resonator to obtain the desired filters and diplexer. The two designed bandpass filters are integrated to form a diplexer. This integration is done with careful consideration of mutual coupling effects and overall system performance. As shown in Fig 4, both BPFs are coupled to a common port (Port 1) and do not have a direct physical connection to each other. BPF1 is coupled to the right side of the thin line connected to the common port and BPF2 is coupled to the left side of this transmission line. Therefore, they do not have loading effect on each other. This means that they do not distort each other’s frequency response. Therefore, the connection is appropriate and desirable.

**Fig 5:**
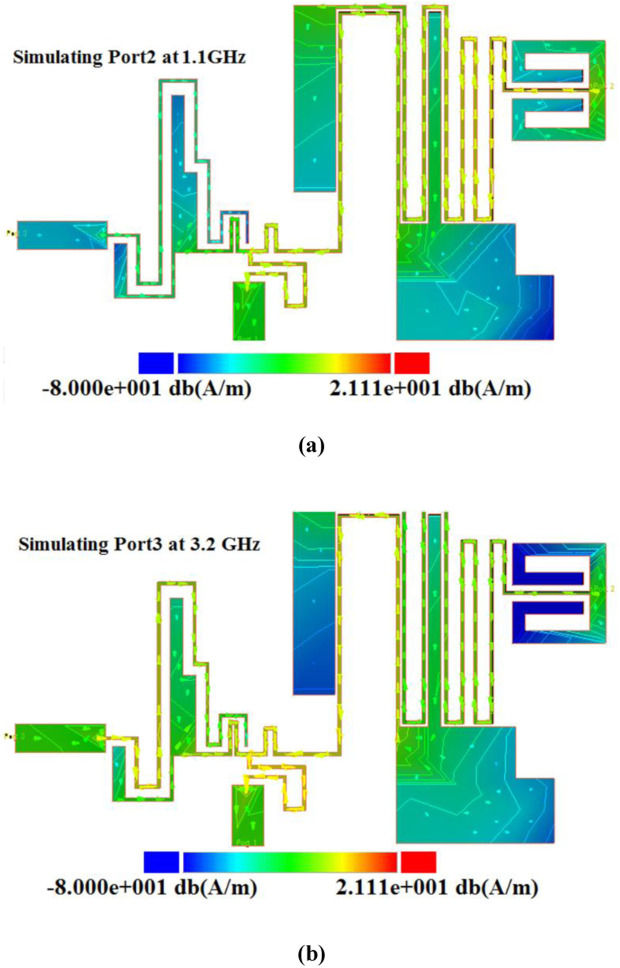
Current density distributions at (a) 1.1 GHz, (b) 3.2 GHz.

Based on the mathematical analysis, the important lengths for optimization are selected and denoted by *l*_*a*_*, l*_*b*_ and *l*_*c*_. The significant widths are selected using the current density distribution in Figs 5(a) and 5(b). Hence, we selected some wide cells with more current density to change and optimize the frequency response. Since after integration, the loading effects of the filters are very small our diplexer didn’t need to an extra matching network. The reason for the low loading effect is using the coupling structures. Ports 2 and 3 are separately coupled to common Port 1, which reduces the effectiveness of Ports 2 and 3.

**Fig 6:**
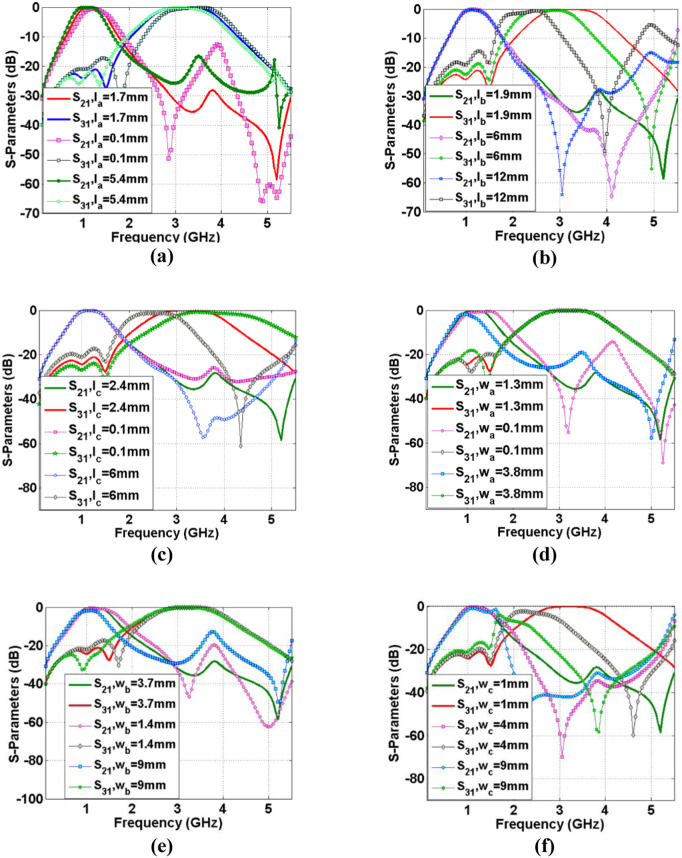
S_21_ and S_31_ of the designed diplexer as functions of (a) l_a_, (b) *l*_*b*_, (c) *l*_*c*_, (d) *w*_*a*_, (e) *w*_*b*_, (f) *w*_*c*_.

**Fig 7:**
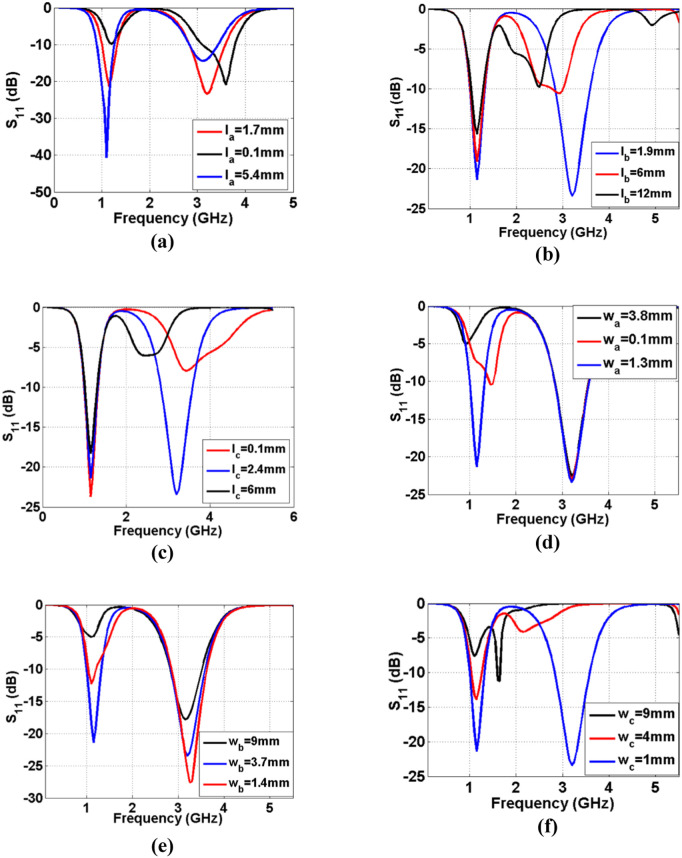
Common port return loss of the designed diplexer as functions of (a) *l*_*a*_, (b) *l*_*b*_, (c) *l*_*c*_, (d) *w*_*a*_, (e) *w*_*b*_, (f) *w*_*c*_.

Figs 6(a)–6(f) depict the frequency response of the introduced diplexer as some functions of the effective lengths and widths. As can be seen in Fig 6(a), increasing and decreasing *l*_*a*_ create some harmonics. Hence, *l*_*a*_* = 1.7 mm* is a reasonable choice. However, by increasing *l*_*a*_ we can improve the return loss. The physical lengths of *l*_*b*_ and *l*_*c*_ affects the second channel (Figs 6(b) and 6(c)), where increasing each of them can shift the resonance frequency to the left. Choosing a neither low nor high value of *l*_*c*_ reduces the return loss at the 2^nd^ channel significantly. As we can see in Figs 6(d) and 6(e), excessive decrease and increase of *w*_*a*_ and *w*_*b*_ will cause harmonics. However, increasing *w*_*c*_ can destroy the 2^nd^ channel completely (see Fig 6(f)). The common port return loss is presented in Figs 7(a)–7(f) as six functions of the significant parameters. As presented in Figs 7(d)–7(f), excessive increase of the widths *w*_*a*_*, w*_*b*_ and *w*_*c*_, lead to increase the return loss in the 1^st^ passband. By enlarging the physical dimensions of the designed diplexer, the operating frequencies move to the left. Increasing the size of patch cells and coupled lines has a greater effect on reducing the operating frequencies. Therefore, by reducing the size of patch stubs and coupled lines, the resonance frequencies will be larger. As depicted in Figs 6(b) and 6(c), by increasing the lengths of coupled lines the second channel is shifted to the left.

The wideband fractional bandwidth observed in this structure is a result of several critical design factors. Firstly, maintaining an optimal size for filled solid cells enhances bandwidth performance by preventing excessive capacitance. However, by excessive decreasing the size of them the operating frequency will be shifted to the left. Secondly, when larger cells are necessary, extending the length of thin connecting lines helps balance the equivalent inductors and capacitors, thereby improving bandwidth. Furthermore, optimizing the structural configuration and coupling between resonators is essential for effective energy transfer, contributing to a broader operational range.

## Simulation and measurement results

**Fig 8:**
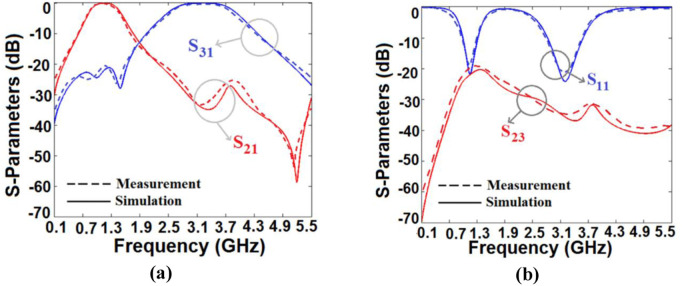
Simulated and measured scattering parameters of our diplexer (a) S_21_ and S_31_, (b) S_11_ and S_23_.

We extracted the simulation results from ADS software using the EM simulator. An HP8757A network analyzer performs the measurements. The simulation and measurement results verify each other. Figs 8(a) and 8(b) depict the measured and simulated scattering parameters of our diplexer. As can be seen, it works at 1.14 GHz and 3.2 GHz, which is appropriate for mid-band 5G applications. The 1^st^ −3 dB passband is from 0.844 GHz to 1.468 GHz and the 2^nd^ is from 2.505 GHz to 3.899 GHz. Therefore, it has wide channels with 57.3% and 44.6% fractional bandwidths. The simulated insertion losses at the 1^st^ and 2^nd^ bands are 0.073 dB and 0.04 dB respectively. Due to having copper loss and some loss in the SMA connectors, the measured losses are slightly higher than the simulated ones. Meanwhile, the return losses at the 1^st^ and 2^nd^ bands are near 21.79 dB and 24.1 dB respectively. As illustrated in Fig 7, the isolation factor up to 5.5 GHz is better than −20.22 dB. The isolation between the channels at the first and second resonance frequencies are −22 dB and −35.7 dB respectively. The simulated and measured harmonics after the first channel are attenuated up to 5.5 GHz. Accordingly, our diplexer suppresses the 1^st^ to 3^rd^ harmonics.

**Fig 9:**
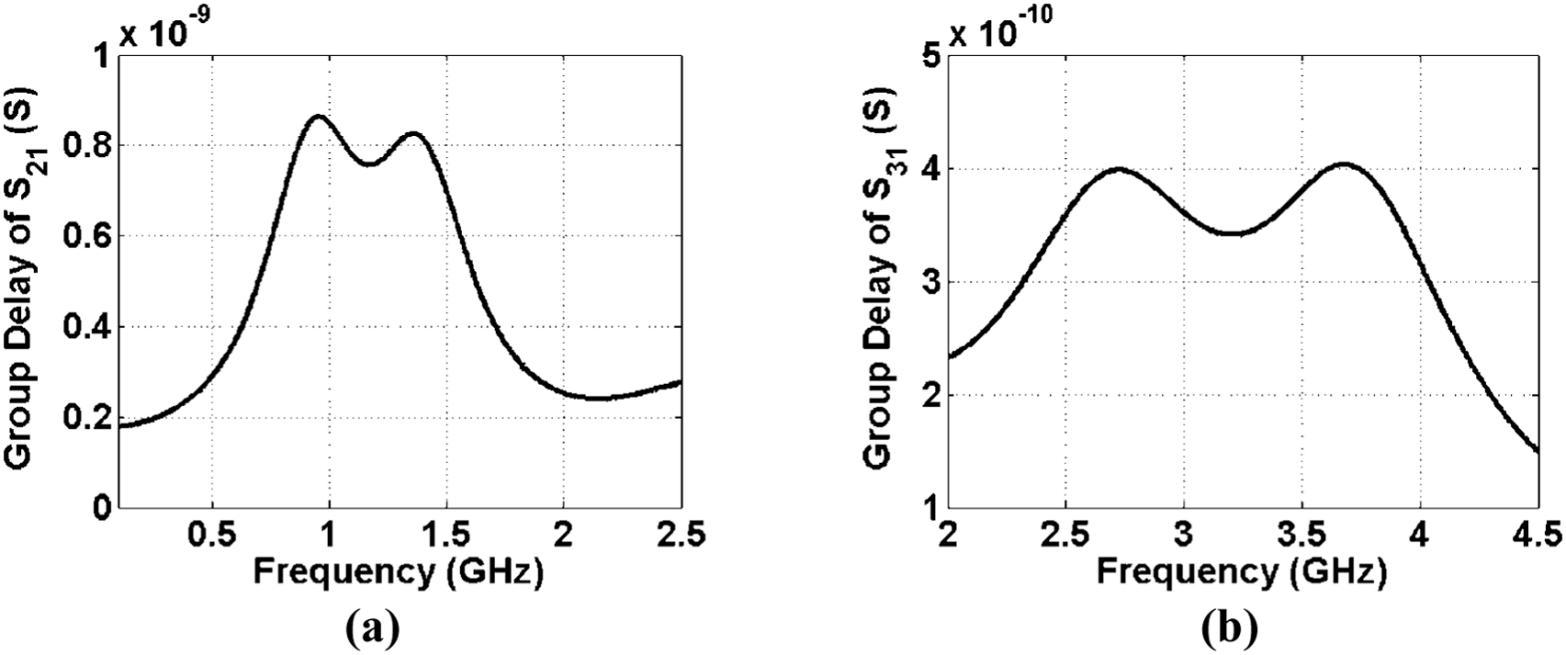
Group delays of (a) S_21_, (b) S_31_.

**Fig 10:**
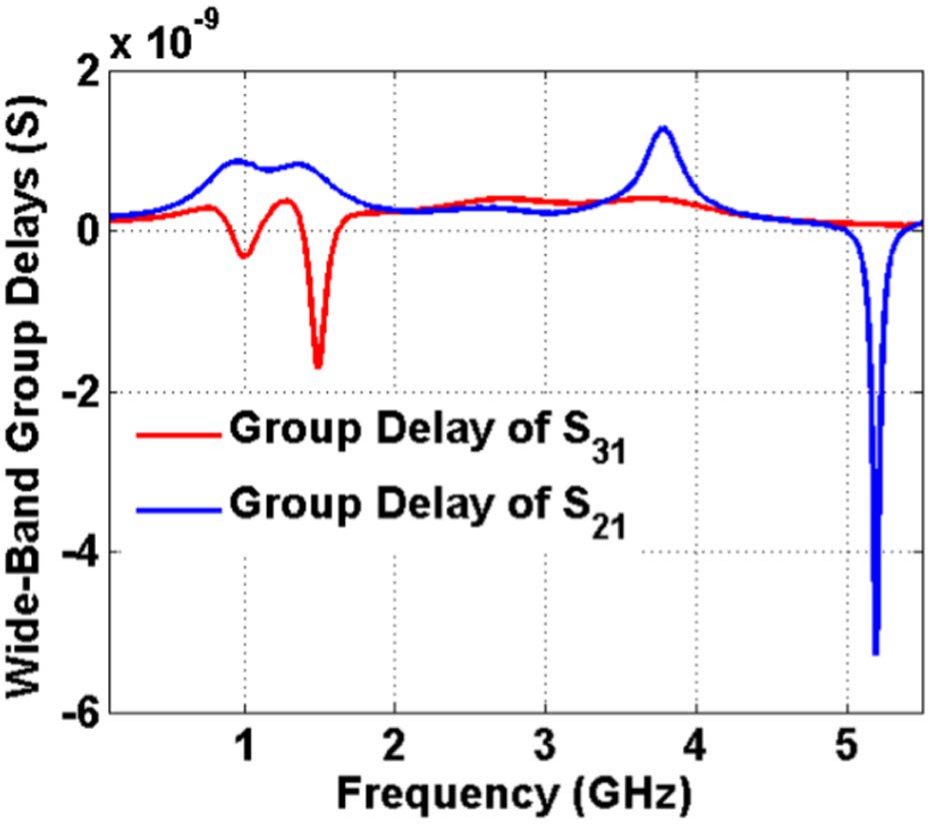
Wide-Band group delays.

**Fig 11:**
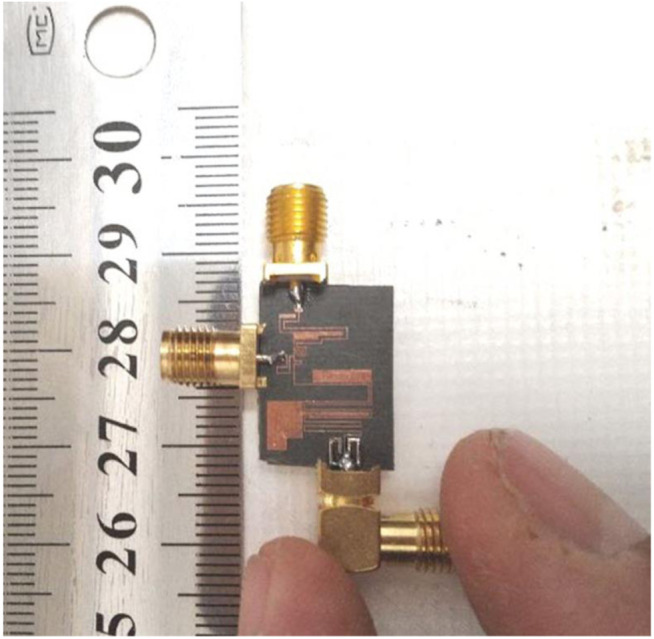
Fabricated diplexer.

Another important parameter of each passive filtering device is the group delay. Usually at a flat channel, the group delay is low. The diplexer group delays (group delays of S_21_ and S_31_) are depicted in Figs 9(a) and 9(b). The group delays at both channels are very low. They are lower than 0.86 ns and 0.4 ns at the first and second bands respectively. Fig 10 shows the wideband group delays of S_21_ and S_31_. It can be seen that it has negative group delays (NGDs) in some frequency ranges. The proposed diplexer exhibits superior group delay performance due to its wide passband and optimal channel spacing. The wide bandwidth facilitates efficient signal transmission, thereby reducing potential distortions that can arise from narrow band filtering. Furthermore, the appropriate separation between channels minimizes interference, leading to a more consistent phase response across the frequency spectrum. As a result, this design achieves a lower group delay variation, which is essential for preserving signal integrity in applications demanding high precision and minimal latency. The group delay of S_31_ is negative from 0.905 GHz to 1.103 GHz. Also, it is negative from 1.38 GHz up to 1.625 GHz. These NGDs can help to mitigate the effects of signal degradation and distortion. Moreover, in systems where bandwidth is a priority, NGD can allow for more efficient use of available spectrum by enabling better separation of channels without interference. Finally, the fabricated diplexer is shown in Fig 11.

## Comparison and discussion

To prove the advantages of this diplexer, we conducted a comparative analysis with the previously reported microstrip diplexers. Table 1 presents the comparison results. As outlined in Table 1, our diplexer demonstrates the lowest insertion loss and the widest fractional bandwidth at the 2^nd^ and 1^st^ channels respectively. Moreover, our diplexer boasts the smallest size compared to the other designs referenced in Table 1. Despite the importance of the delay group, unfortunately, most bandpass-bandpass diplexer designers have not paid attention to it. Therefore, we had to compare it with other microstrip filtering devices to prove that our group delays are low. Accordingly, in Table 2 our group delays (the maximum values in each band) are compared with the previous works. As noted, in general we could improve the group delays compared to the previous work. Only at the first band of Lowpass-Bandpass (LP-BP) diplexer in [31] the group delay is a little better than our bandpass-bandpass design. However, we could improve it in the upper channel where at the 2^nd^ channel, group delays of reference [31] and our diplexer are 2.5 ns and 0.4 ns respectively. Our proposed design has a completely novel microstrip structure, where it is presented for the first time in this work. Having an ultra-compact structure, the minimum losses, the widest FBWs and the lowest group delay confirms the high efficiency of this diplexer. In conclusion, the presented diplexer demonstrates superior performance compared to previous microstrip diplexers, making it a highly promising solution for various communication applications. This diplexer is designed for wideband applications. Since both passbands are wide, the channels are close. This leads to decrease the isolation between the channels. However, the isolation between channels of this diplexer is acceptable.

**Table 1:** Comparison (FBW: Fractional Bandwidth; RL: Return Loss; IL: Insertion Loss).

Refs.	IL_1_ and IL_2_ (dB)	RL_1_ and RL_2_ (dB)	FBW_1_ and FBW_2_ (%)	Size (λg^2^)
This Diplexer	0.073, 0.04	21.79, 24.1	57.3, 44.6	0.004
[14]	0.7, 0.9	21.2, 17	1.41, 2.2	---
[15]	1.3, 1.36	19, 17	2.5, 2.3	---
[16]	0.3, 0.4	10, 18	14, 4	0.083
[17]	1.6, 2.6	15.6, 15	---	---
[18]	1.1, 1.2	35, 45	3.2, 3	0.26
[19]	1.4, 2.3	15, 20	6.1, 4	0.089
[20]	1, 0.9	20, 20	6.1, 5.8	0.127
[21]	0.87, 1.25	17, 15.5	21.2, 13.2	0.12
[22]	0.5, 0.2	12, 20	---	0.04
[23]	0.21, 0.21	32, 25	4.6, 4.6	0.018
[24]	1.45, 1.55	13, 30	5.9, 8.8	0.029
[25]	0.7, 0.5	19.6, 22	---	0.017
[26]	4.2, 3.3	32, 31	---	---

**Table 2:** Maximum group delays comparison (*: Approx.).

References	Type	Maximum group delays at each channel
This work	BP-BP Diplexer	0.86 ns, 0.4 ns
[14]	BP-BP Diplexer	Near 4 ns*, near 3 ns*
[29]	LP-BP Triplexer	1.62 ns, 1.75 ns, 2.07 ns
[30]	LP-BP Diplexer	1.43 ns, 1.68 ns
[31]	LP-BP Diplexer	0.65 ns *, more than 2.5 ns *
[32]	Tri-Channel Bandpass Filter	Better than 8 ns at all channels
[33]	LP-BP Triplexer	1.5 ns, 6 ns, 4.4 ns
[34]	Quad-Channel BP-BP Diplexer	2.76 ns, 3.31 ns, 0.91 ns, 2.15 ns
[35]	Quad-Channel Bandpass Filters	9 ns, 6 ns, 6 ns, 5 ns

## Future works

Although the presented diplexer design offers high performance and compact size future research can delve into exploring various design parameters, such as the dimensions and shapes of the microstrip elements, to improve the overall performance. Additionally, the choice of materials employed in the diplexer can significantly affect its performance. Future studies can explore the use of alternative materials with improved electrical properties, such as low dielectric loss and high thermal stability, to further enhance the performance characteristics of the diplexer. The diplexer can be further integrated into larger communication systems by exploring its compatibility with other components, such as amplifiers, filters, and antennas. Investigating the interaction between the diplexer and these components can lead to the progress of more efficient and reliable communication systems. Furthermore, it is crucial to evaluate the diplexer’s performance in various real-world scenarios to assess its robustness and reliability. Subsequent research may concentrate on testing the diplexer under different environmental conditions, such as temperature variations, humidity, and exposure to electromagnetic interference, to validate its suitability for practical applications.

## Conclusion

This paper presents a comprehensive study of the design, analysis, simulation, and fabrication of a high-performance diplexer with a novel microstrip configuration. It operates at 1.1 GHz and 3.2 GHz for wide-band and mid-band 5G applications. The proposed diplexer integrates spiral-coupled lines and filled rectangular cells. The diplexer exhibits a compact size, novel structure, low losses, wide bands, and close agreement between the experimental results and simulations. The compact size of this diplexer is achieved through careful design and optimization techniques. We analyzed the basic structure mathematically to improve the performance and miniaturization. Comparative analysis with previous works referred in this study reveals low insertion losses (0.07 dB, 0.04 dB), low group delays, wide fractional bandwidths, making it the most compact among its counterparts. Meanwhile, the return losses (RLs) at the 1^st^ and 2^nd^ bands are 21.79 dB and 24.1 dB respectively. After the lower channel the 1^st^ to 3^rd^ harmonics are suppressed. The experimental results demonstrate close agreement between the measured and simulated performance of our diplexer, validating the effectiveness of the design approach. This confirms the suitability of this diplexer for 5G applications.
